# Cancer cell-derived exosomal circUHRF1 induces natural killer cell exhaustion and may cause resistance to anti-PD1 therapy in hepatocellular carcinoma

**DOI:** 10.1186/s12943-020-01222-5

**Published:** 2020-06-27

**Authors:** Peng-Fei Zhang, Chao Gao, Xiao-Yong Huang, Jia-Cheng Lu, Xiao-Jun Guo, Guo-Ming Shi, Jia-Bin Cai, Ai-Wu Ke

**Affiliations:** 1 Liver Cancer Institute, Zhongshan Hospital of Fudan University, 180 Fenglin Road, Shanghai, People’s Republic of China 200032; 2 Key Laboratory of Carcinogenesis and Cancer Invasion, Ministry of Education, Shanghai, 200032 People’s Republic of China; 3 Department of Medical Oncology, Zhongshan Hospital, Fudan University, Shanghai, 200032 P.R. China

## Abstract

**Objective:**

Natural killer (NK) cells play a critical role in the innate antitumor immune response. Recently, NK cell dysfunction has been verified in various malignant tumors, including hepatocellular carcinoma (HCC). However, the molecular biological mechanisms of NK cell dysfunction in human HCC are still obscure.

**Methods:**

The expression of circular ubiquitin-like with PHD and ring finger domain 1 RNA (circUHRF1) in HCC tissues, exosomes, and cell lines was detected by qRT-PCR. Exosomes were isolated from the culture medium of HCC cells and plasma of HCC patients using an ultracentrifugation method and the ExoQuick Exosome Precipitation Solution kit and then characterized by transmission electronic microscopy, NanoSight and western blotting. The role of circUHRF1 in NK cell dysfunction was assessed by ELISA. In vivo circRNA precipitation, RNA immunoprecipitation, and luciferase reporter assays were performed to explore the molecular mechanisms of circUHRF1 in NK cells. In a retrospective study, the clinical characteristics and prognostic significance of circUHRF1 were determined in HCC tissues.

**Results:**

Here, we report that the expression of circUHRF1 is higher in human HCC tissues than in matched adjacent nontumor tissues. Increased levels of circUHRF1 indicate poor clinical prognosis and NK cell dysfunction in patients with HCC. In HCC patient plasma, circUHRF1 is predominantly secreted by HCC cells in an exosomal manner, and circUHRF1 inhibits NK cell-derived IFN-γ and TNF-α secretion. A high level of plasma exosomal circUHRF1 is associated with a decreased NK cell proportion and decreased NK cell tumor infiltration. Moreover, circUHRF1 inhibits NK cell function by upregulating the expression of TIM-3 via degradation of miR-449c-5p. Finally, we show that circUHRF1 may drive resistance to anti-PD1 immunotherapy in HCC patients.

**Conclusions:**

Exosomal circUHRF1 is predominantly secreted by HCC cells and contributes to immunosuppression by inducing NK cell dysfunction in HCC. CircUHRF1 may drive resistance to anti-PD1 immunotherapy, providing a potential therapeutic strategy for patients with HCC.

## Introduction

Hepatocellular carcinoma (HCC) is the fifth most common cancer and the second leading cause of cancer death in the world [1]. However, despite the rapid advancements in diagnosis, surgical techniques, targeted therapy, and immunotherapy, the 5-year overall survival rate of HCC patients remains unsatisfactory due to relapse with distant metastasis and resistance to antitumor agents [2–4]. The underlying biological molecular mechanisms of HCC tumorigenesis, metastasis, and resistance to anti-HCC agents remain obscure [5–7]. Therefore, further exploration of HCC tumorigenesis and progression mechanisms will provide new promising therapeutic strategies for HCC.

T cell immunoglobulin and mucin domain 3 (TIM-3) is an immunomodulatory receptor that engages with ligands on tumor cells and the microenvironment to inhibit antitumoral immunity in a variety of cancers, including HCC [8–10]. TIM-3 is one of the major inhibitory receptors on natural killer (NK) cells, and NK cells with forced TIM-3 expression have a reduced ability to mediate antitumoral immunity [11]. Furthermore, blockade of TIM-3 may represent a novel strategy to increase NK function in cancer patients [11]. In addition, a higher density of tumoral NK cells is associated with a response to anti-PD1 therapy in tumors [12, 13]. Importantly, a previous study reported that increased TIM-3 expression was detected in NK-92 cells transfected with an HBV expression vector and NK cells isolated from the livers of HBV transgenic mice [10]. Moreover, blockade of TIM-3 resulted in increased cytotoxicity of NK cells against HCC cells, as well as increased interferon-gamma (IFN-γ) production [10]. However, research on NK cells in HCC has been relatively scarce despite considerable evidence showing that they have an important role in malignancy.

Ubiquitin-like with PHD and RING finger domain 1 (UHRF1) is a critical molecule that participates in regulating DNA methylation and is usually overexpressed in many cancers, including HCC [14]. Importantly, forced UHRF1 expression promotes HCC tumorigenesis and progression [14]. Therefore, we speculated that UHRF1-derived circRNA expression might be upregulated and might promote the progression of HCC. Here, we analyzed UHRF1-derived circRNA expression profiles in human HCC tissues, adjacent nontumor tissues, and HCC-derived exosomes and identified circUHRF1 (hsa_circ_0048677) as a significantly increased circRNA in HCC tissues. Furthermore, the expression of circUHRF1 was closely related to poor prognosis in HCC patients. Additionally, we found that HCC-derived exosomal circUHRF1 upregulates the expression of the miR-449c-5p target gene TIM-3 in NK cells by degrading miR-449c-5p, thereby promoting immune evasion and resistance to anti-PD1 immunotherapy in HCC. Thus, circUHRF1 might act as a promising therapeutic target in HCC patients.

## Methods

### Cell lines and clinical tissues

Six human HCC cell lines (HepG2, HCCLM3, SMMC-7721, Huh 7, PLC/PRF/5, and Hep3B) were cultured in Dulbecco’s modified Eagle’s medium (DMEM, HyClone, Cat: SH30243) supplemented with 10% fetal bovine serum (FBS, Gibco, Cat: 10100147). The NK-92 cell line was cultured in RPMI-1640 (HyClone, Cat: SH30809) supplemented with 20% FBS and 150 IU/mL recombinant human interleukin-2 (IL-2) (Novoprotein, Shanghai, Cat: GMP-C013). The K562 cell line was cultured in RPMI-1640 supplemented with 10% FBS. All of the above cell lines were cultured at 37 °C in a 5% CO_2_ incubator.

The tissue samples used in this study were collected as described in Additional file 1**: Supplementary Materials and Methods**.

### Exosome isolation and electron microscopy

Exosomes from the serum of HCC patients and culture medium of HCC cells were isolated using ExoQuick Exosome Precipitation Solution (SBI System Biosciences, Cat: EXOQ5A-1) according to the manufacturer’s instructions. Then, the exosomes were examined by transmission electron microscopy as described previously [15–17].

### Quantitative real-time polymerase chain reaction (qRT-PCR) analysis and western blotting analysis

qRT-PCR and western blotting analyses were performed as described previously and in Additional file 1**: Supplementary Materials and Methods** [18]. The primers and antibodies used in this study are listed in Additional file 2**: Supplementary Tables 1 and 2**.

### Immunohistochemistry

Immunohistochemistry was performed, and the intensity of the positive staining was measured as described in our previous study [18]. The anti-NKG2D antibody used in this study is listed in Additional file 2**: Supplementary Table 2**.

### RNA immunoprecipitation (RIP)

RIP assays were performed using a Magna RIP RNA Binding Protein Immunoprecipitation Kit (Millipore, Cat: 17–770) according to the manufacturer’s instructions. The anti-Argonaute 2 (AGO2) and IgG antibodies used in this study are listed in Additional file 2**: Supplementary Table 2**.

### In vivo circRNA precipitation (circRIP) assay

Biotin-labeled circUHRF1 and negative control probes (Supplementary Table 3) were synthesized by GenePharma (Shanghai, China). The circRIP assay was performed as described previously [18]. The sequence of the circUHRF1 probe is listed in Additional file 2**: Supplementary Table 3**.

### Luciferase reporter assay

The wild-type TIM-3 3′ UTR and circUHRF1 sequences were cloned into a pLG3 plasmid. The mutant TIM-3 3′ UTR and circUHRF1 pLG3 plasmids were generated using a mutagenesis kit (Qiagen, USA, Cat: 200521) according to the manufacturer’s instructions. Healthy donor-derived NK cells and NK-92 cells were seeded into 96-well plates and cotransfected with a luciferase reporter vector and miR-449c-5p mimics or the negative control using the Lipofectamine 2000 transfection reagent (Thermo Fisher, Cat: 11668–019). After 48 h, the firefly and Renilla luciferase activities were quantified with the Dual-Luciferase Reporter Assay System (Promega, USA, Cat: E1910).

### Preparation of purified NK cells and CD8^+^ T cells

NK cells (CD16^+^/CD56^+^) (Miltenyi, Cat: 130–092-660) and CD8^+^ T cells (Miltenyi, Cat: 130–045-201) were obtained from peripheral blood mononuclear cells (PBMCs) of healthy donors by positive selection using magnetic cell separation according to the manufacturer’s instructions.

### Enzyme linked immunosorbent assay (ELISA)

The concentrations of IFN-γ and TNF-α produced by NK cells and CD8^+^ T cells were measured by IFN-γ and TNF-α ELISA kits (eBioscience, USA, Cat: KHC4021 and Cat: BMS223HS) in accordance with the manufacturer’s guidelines.

### RNA pulldown assay

The pulldown assay was performed as described previously [19, 20]. In brief, to generate probe-coated beads, the biotinylated circUHRF1 probe, biotinylated circANRIL probe, and biotinylated negative control (NC) probe (GenePharma, China) were incubated with M-280 streptavidin magnetic beads (Invitrogen, USA, Cat: 11205D) at room temperature for 2 h. Then, approximately 1 × 10^7^ NK-92 cells were harvested, lysed, sonicated and incubated with probe-coated beads at 4 °C overnight. Subsequently, the RNA complexes bound to the beads were eluted and extracted with an RNeasy Mini Kit (QIAGEN, USA, Cat: 74104) and analyzed by qRT-PCR assay.

### In vivo metastasis assays

The in vivo metastasis assays are described in our previous studies [21] and in the **Supplementary Materials and Methods**.

### Transfection experiment

All lentiviral vectors used in this study were purchased from Genomeditech (Shanghai, China). The circUHRF1-overexpression and circUHRF1-shRNA lentiviral vectors were transfected into NK-92 cells, and the blank lentiviral vectors were used as negative controls. The circUHRF1 shRNA target sequences used in this study are listed in Additional file 2**: Supplementary Table 4**.

### In vivo anti-PD1 experiments

Male NOD/SCID mice aged 4–6 weeks were maintained according to the stated guidelines. HCCLM3 cells (5 × 10^6^) with or without reduced circUHRF1 were injected into the mouse right flank to generate subcutaneous tumors. When, the tumor reached a volume of approximately 100 mm^3^, NK cells (1 × 10^6^) were resuspended in phosphate-buffered saline and injected intravenously through the tail vein. The mice were randomly assigned to four groups. Then, the mice received tail vein injection of Opdivo or its isotype control at 100 μg per dose three times a week for 2 weeks. Animals were euthanized when tumors reached a maximum of 1000 mm^3^ (*n* = 6). The day that the mice received the first therapy was considered day 1. Tumor volume was calculated as (length x width^2^)/2.

### Statistical analysis

Statistical analyses were performed with SPSS software (19.0; SPSS, Inc., Chicago, IL). Values are presented as the mean ± standard deviation (SD). The statistical analyses are described in detail in the **Supplementary Materials and Methods**. *P* < 0.05 was considered statistically significant.

## Results

### Analysis of UHRF1-related circRNAs in HCC tissues

**Fig. 1:**
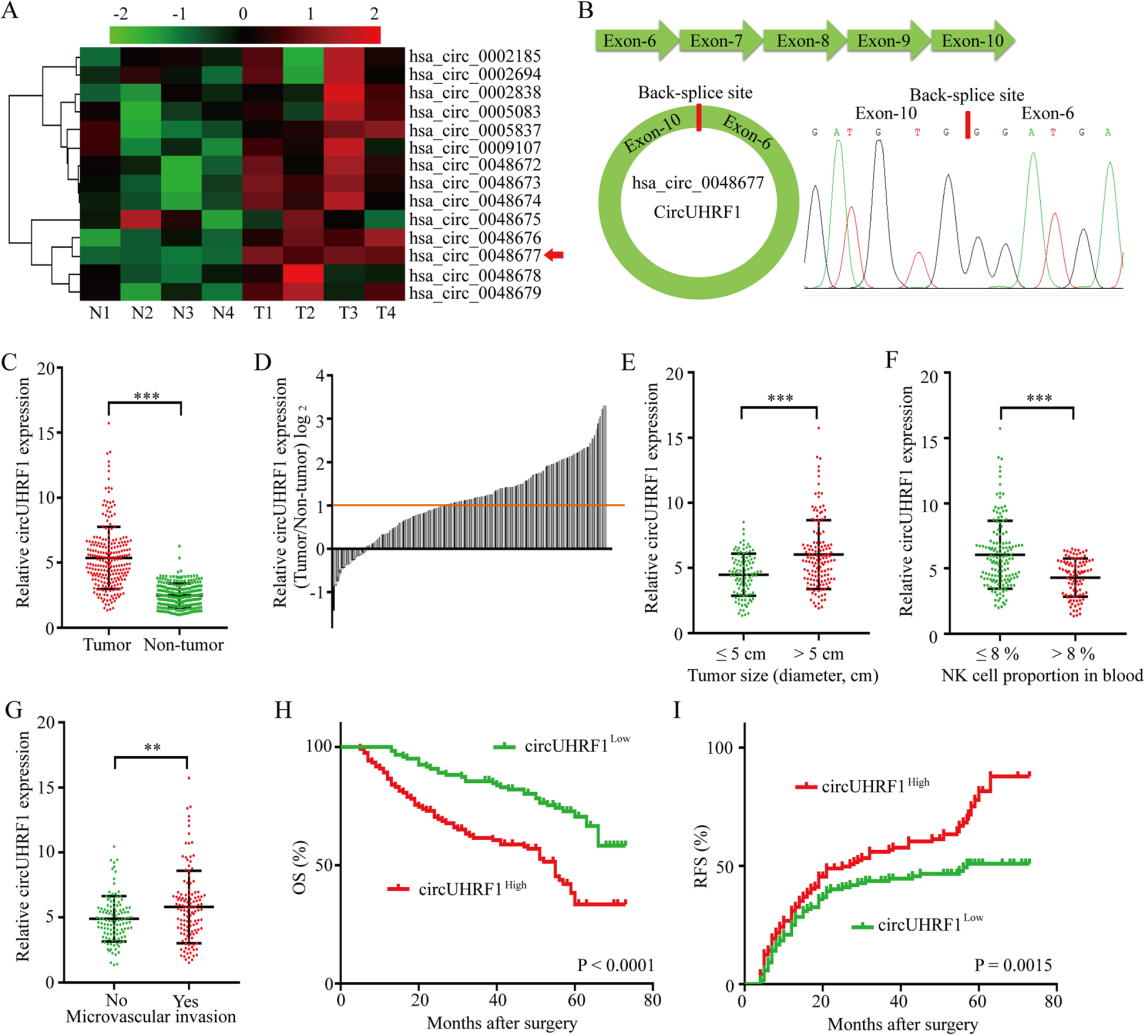
CircUHRF1 is upregulated in HCC tissues. **a** Heatmap showing UHRF1 gene-derived circRNAs in HCC tissues compared with matched adjacent nontumor tissues analyzed by qRT-PCR. **b** Schematic illustration of circUHRF1. **c** Differential expression of circUHRF1 in HCC tissues and adjacent nontumor tissues of 240 patients. **d** CircUHRF1 levels in 240 pairs of HCC and matched adjacent nontumor tissues, shown as log_2_ (T/N). **e** A total of 240 patients were divided into ≤5 cm and > 5 cm size groups. The diagram shows circUHRF1 expression in each group. **f** A total of 240 patients were divided into groups based on NK cell proportion in the blood (≤ 8% and > 8%). The diagram shows circUHRF1 expression in each group. **g** A total of 240 patients were divided into groups with or without microvascular invasion. The diagram shows circUHRF1 expression in each group. **h and i** Kaplan-Meier analysis of overall survival and recurrence in 240 patients with HCC according to circUHRF1 expression (log-rank test). Data are presented as the means ± SD of three independent experiments. **P < 0.01, ***P < 0.001

UHRF1 is frequently upregulated and plays a critical pathological role in HCC progression [14]. Furthermore, circRNAs were found to be produced from the back-splicing of precursor mRNAs (pre-mRNAs) and are considered byproducts of pre-mRNA splicing in cancer cells [22–24]. Usually, the levels of circRNAs are in accordance with their respective mRNA levels [25]. Therefore, we examined the levels of 14 circular RNAs derived from UHRF1 by qRT-PCR (circular RNA sequencing data were obtained from the online database circBase). Among them, hsa_circ_0048677 (circUHRF1) was the most abundant circRNA and was upregulated in HCC tissues (Fig. 1a and b). Then, we examined the expression of circUHRF1 by qRT-PCR in 240 HCC tissues and matched adjacent nontumor tissues. CircUHRF1 expression in HCC tissues was significantly higher than that in adjacent nontumor tissues (tumor/adjacent nontumor tissues ≥2; 136/240) (Fig. 1c and d). Next, we explored the relationship between circUHRF1 expression and the clinicopathological characteristics of 240 HCC patients, and the results are listed in Table 1. The results showed that HCC patients with circUHRF1^high^ expression had larger tumor size (*P* = 0.001) (Fig. 1e), lower NK cell proportions in blood (*P* = 0.005) (Fig. 1f), and more microvascular invasion (*P* = 0.020) (Fig. 1g) than those with circUHRF1^low^ expression. Furthermore, we analyzed the prognostic potential of circUHRF1 expression in HCC patients. Importantly, Kaplan-Meier survival analysis indicated that patients with circUHRF1^high^ expression levels had a poor clinical prognosis (Fig. 1h and i). Multivariate analysis identified circUHRF1 expression as an independent predictor of overall survival (OS) and postoperative recurrence (Tables 2 and 3). In addition, we also analyzed the clinical data in the hepatitis B-positive patients (216/240) and found that the findings in hepatitis B-positive patients alone were consistent with those in overall patient population, which might be due to the large proportion of hepatitis B-positive patients in this cohort. Taken together, our data suggest that upregulated circUHRF1 expression in HCC cells is correlated with poor outcome in patients and indicate that increased circUHRF1 expression is likely involved in the progression of HCC.

**Table 1:** Correlation between circUHRF1 and clinicopathological characteristics in 240 HCC patients

Variable	circUHRF1		*P* value
	Low	High	
Age (years)			
≤ 50	52	57	0.604
> 50	68	63	
Sex			
Female	41	37	0.679
Male	79	83	
HBsAg			
Negative	15	9	0.282
Positive	105	111	
HCVAb			
Negative	101	107	0.343
Positive	19	13	
Liver cirrhosis			
No	71	62	0.299
Yes	49	58	
Serum AFP, ng/mL			
≤ 20	8	17	0.089
> 20	112	103	
Serum ALT, U/L			
≤ 75	88	75	0.097
> 75	32	45	
Tumor size (diameter, cm)			
≤ 5	65	38	0.001
> 5	55	82	
NK cell proportion in blood			
≤ 8%	62	84	0.005
> 8%	58	36	
Microvascular invasion			
No	67	48	0.020
Yes	53	72	
Tumor number			
Single	84	76	0.338
Multiple	36	44	
TNM			
I/II	44	52	0.356
III/IV	76	68

**Table 2:** Univariate and multivariate analyses of factors associated with overall survival

	OS			
		Multivariate		
Factors	Univariate, P	HR	95% CI	*P* value
Sex (Female vs. Male)	0.771			NA
Age (years) (≤50 vs. > 50)	0.417			NA
HBsAg (Positive vs. Negative)	0.274			NA
HCVAb (Positive vs. Negative)	0.622			NA
Liver cirrhosis (Yes vs. No)	0.318			NA
Serum AFP, ng/mL (≤20 vs. > 20)	0.629			NA
Serum ALT, U/L (≤75 vs. > 75)	0.811			NA
Tumor size (diameter, cm) (> 5 vs ≤ 5)	0.036	1.622	1.107–2.514	NS
NK cell proportion in blood (≤ 8% vs. > 8%)	0.204			NA
TNM (III/IV vs I/II.)	0.071			NA
CircUHRF1 expression (High vs. Low)	0.003	1.339	0.944–2.045	0.022

*OS* overall survival; *NA* not adopted; *AFP* alpha-fetoprotein; *HBsAg* hepatitis B surface antigen; *95%CI* 95% confidence interval; *HR* hazard ratio; Cox proportional hazards regression model

**Table 3:** Univariate and multivariate analyses of factors associated with cumulative recurrence

	Cumulative Recurrence			
		Multivariate		
Factors	Univariate, P	HR	95% CI	*P* value
Sex (Female vs. Male)	0.527			NA
Age (years) (≤50 vs. > 50)	0.393			NA
HBsAg (Positive vs. Negative)	0.266			NA
HCVAb (Positive vs. Negative)	0.494			NA
Liver cirrhosis (Yes vs. No)	0.802			NA
Serum AFP, ng/mL (≤20 vs. > 20)	0.472			NA
Serum ALT, U/L (≤75 vs. > 75)	0.615			NA
Tumor size (diameter, cm) (> 5 vs ≤ 5)	0.066			NA
NK cell proportion in blood (≤ 8% vs. > 8%)	0.149			NA
TNM (III/IV vs I/II.)	0.277			NA
CircUHRF1 expression (High vs. Low)	0.004	1.762	1.172–2.428	0.019

*NA* not adopted; *AFP* alpha-fetoprotein; *HBsAg* hepatitis B surface antigen; *95%CI* 95% confidence interval; *BCLC* Barcelona-Clinic Liver Cancer, *HR* hazard ratio; Cox proportional hazards regression model

### HCC cells secrete circUHRF1 in an exosomal manner

**Fig. 2:**
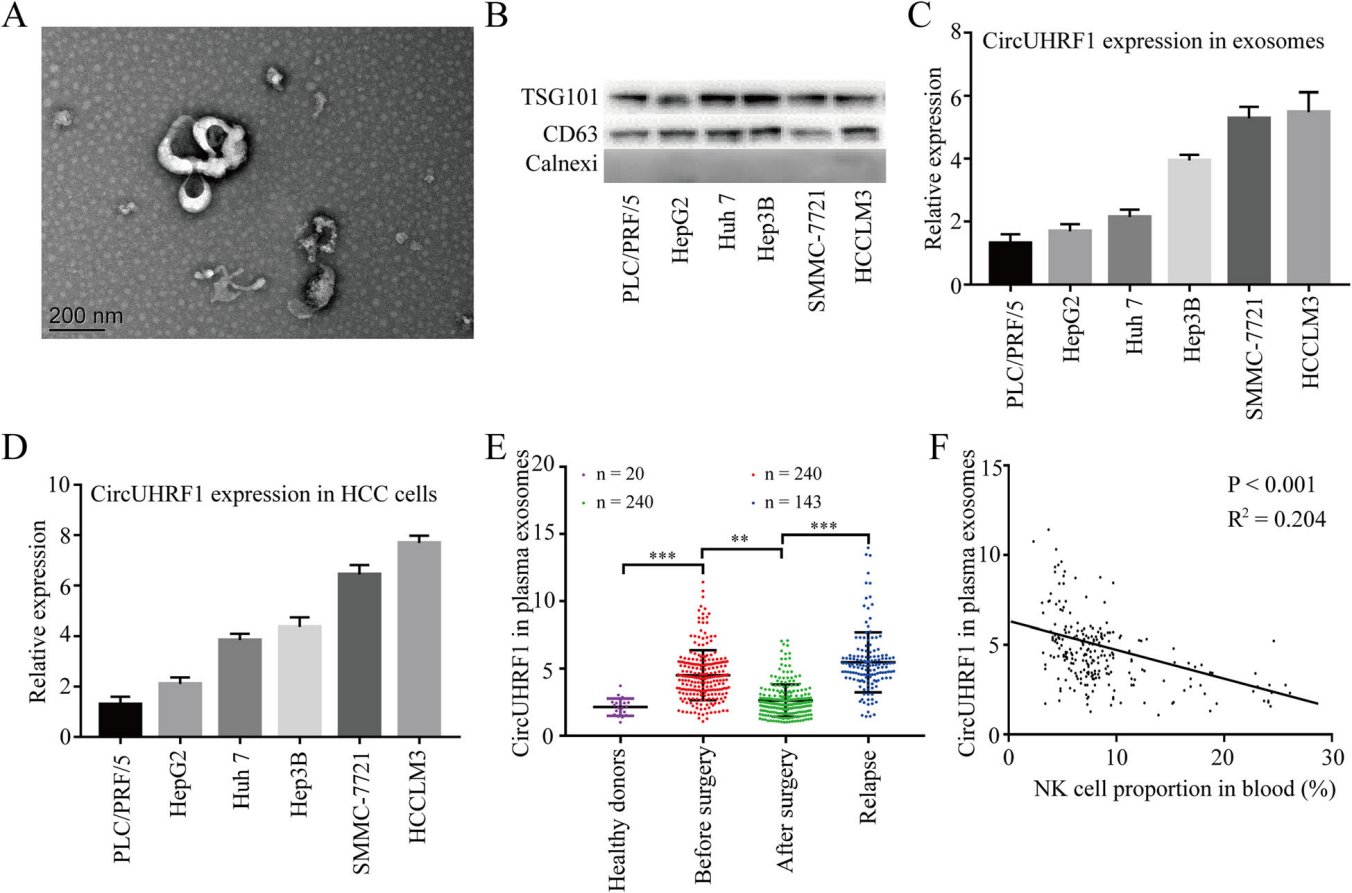
Characterization of exosomes and the expression of circUHRF1. **a** High-resolution transmission electron microscopy image of HCC cell exosomes (scale bar = 200 nm). **b** The expression of exosomal biomarkers in HCC cell-derived exosomes was detected by western blot. **c** CircUHRF1 expression in HCC cell-derived exosomes was measured by qRT-PCR analysis. **d** circUHRF1 expression in HCC cells was measured by qRT-PCR analysis. **e** Exosomal circUHRF1 expression in the serum of healthy donors, patients before surgery, patients after surgery, and patients with recurrence. **f** A negative correlation between circUHRF1 expression in plasma exosomes and the proportion of NK cells was observed in HCC patient blood (*R*^2^ = 0.2040; *P* < 0.001). The data are presented as the mean ± SD; *n* = 3, *P < 0.05, **P < 0.01

Previous studies have reported that circRNA can be transferred from cell to cell via exosomes [26, 27]. Thus, we hypothesized that circUHRF1 could be transferred from HCC cells to other cells. To verify this hypothesis, exosomes from the supernatants of HCC cell lines were isolated and evaluated by TEM and western blotting (Fig. 2a and b). Then, qRT-PCR analyses in six HCC cell lines and paired exosomes showed that circUHRF1 was markedly upregulated in the HCCLM3 and SMMC-7721 cell lines compared with the other cell lines (Fig. 2c). Interestingly, circUHRF1 expression in exosomes was consistent with the parental cell expression levels (Fig. 2d).

Because exosomal circUHRF1 was detected in HCC-derived exosomes, we explored whether circUHRF1 was present in HCC patient plasma. Importantly, circUHRF1 was present in plasma exosomal RNA. Plasma exosomal circUHRF1 levels were increased in HCC patients compared with 20 healthy donors (Fig. 2e). Furthermore, plasma exosomal circUHRF1 levels were reduced after tumor resection and increased in patients with tumor relapse, indicating that plasma exosomal circUHRF1 was mainly produced by HCC cells (Fig. 2e). Interestingly, exosomal circUHRF1 levels were significantly increased in the plasma of patients with evidence of immune evasion mechanisms, which had a decreased NK cell proportion in peripheral circulation (Fig. 2f). These data suggested that plasma exosomal circUHRF1 could serve as a critical molecular determinant of NK cell-related immune evasion.

### HCC cells suppress NK cell functions through exosomal circUHRF1

**Fig. 3:**
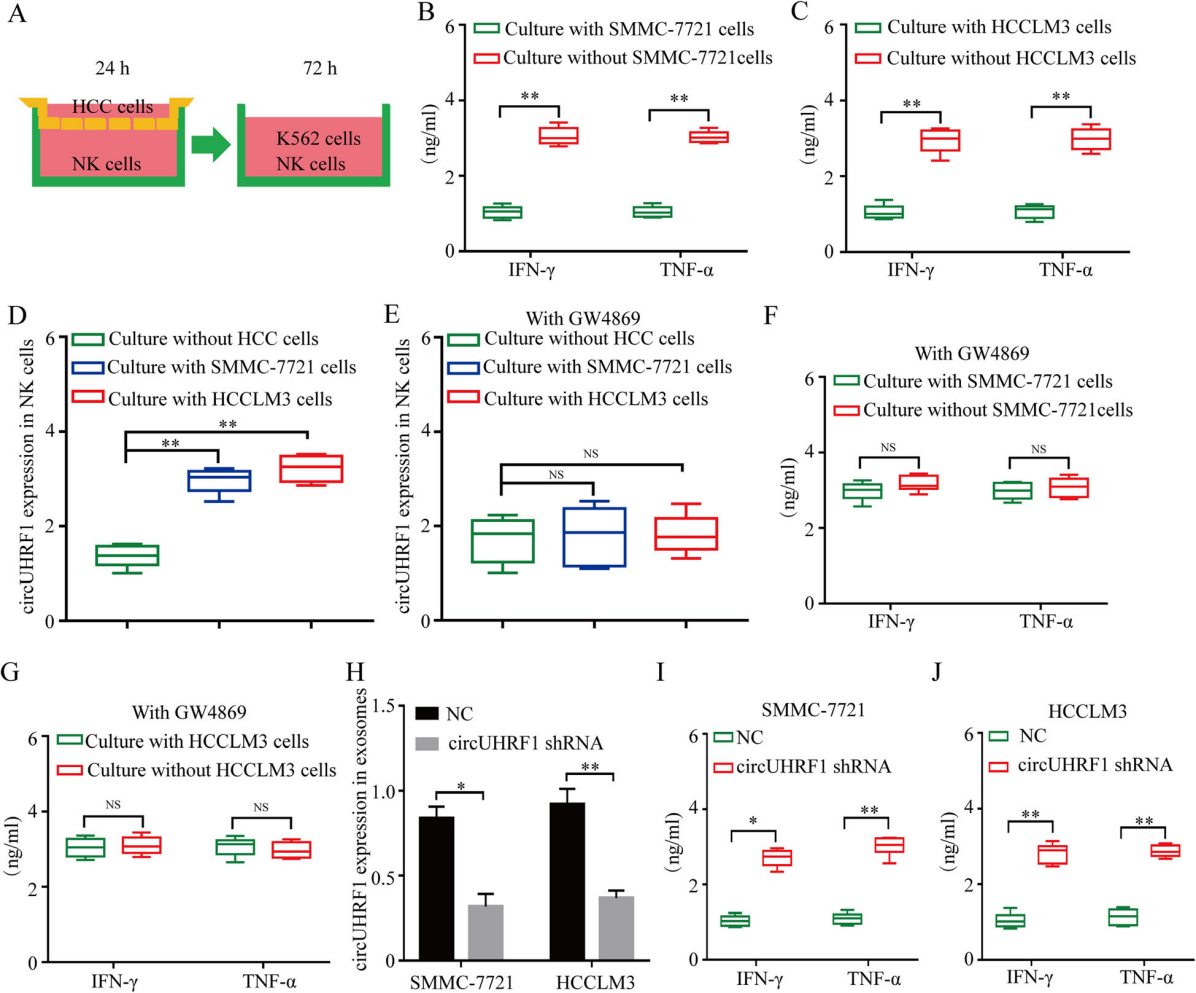
Exosomal expression of circUHRF1 inhibits the function of NK cells. **a** Schematic diagram of the coculture model. **b** and **c** HCC cells inhibit NK cell secretion of IFN-γ and TNF-α. **d** HCC cells upregulate the expression of circUHRF1 in NK cells. **e** GW4869 inhibits HCC cell-induced circUHRF1 upregulation in NK cells. **f and g** The effect of GW4869 on HCC-induced IFN-γ and TNF-α secretion by NK cells. **h** CircUHRF1 silencing is accompanied by decreased exosomal circUHRF1. **i** and **j** Decreased exosomal circUHRF1 promotes HCC-induced IFN-γ and TNF-α secretion by NK cells. The data are presented as the mean ± SD; *n* = 3, *P < 0.05, **P < 0.01, NS: not significant

To determine whether HCC-derived exosomal circUHRF1 induces alterations in allogeneic NK cells, NK cells from 6 healthy donors were cocultured with HCCLM3 and SMMC-7721 cells at a 1:1 ratio for 24 h (Fig. 3a). Compared with NK cells cultured alone, NK cells cocultured with SMMC-7721 and HCCLM3 cells demonstrated significant impairment of IFN-γ and TNF-α secretion when subsequently cocultured with K562 cells (Fig. 3a-c). In addition, NK cells cocultured with SMMC-7721 and HCCLM3 cells demonstrated significantly increased circUHRF1 expression (Fig. 3d), which was impaired by the exosome inhibitor GW4869 (Fig. 3e). NK cells cocultured with SMMC-7721 and HCCLM3 cells prior to treatment with an exosome inhibitor exhibited attenuated impairment of IFN-γ and TNF-α secretion when subsequently cocultured with K562 cells (Fig. 3f and g). To further investigate whether the inhibitory effects of HCC cells on NK cell function were mediated in an exosomal circUHRF1-dependent manner, circUHRF1 knockdown cells were established using an shRNA lentiviral vector (which targeted the back-splice site of circUHRF1) in SMMC-7721 and HCCLM3 cells (Additional file 3**: Supplementary Fig. 1a**). To further verify that circUHRF1 shRNA had no effects on other UHRF1 splicing products, qRT-PCR was carried out to detect UHRF1 mRNA expression. The expression of UHRF1 mRNA showed no significant change after transfection with circUHRF1 shRNA, indicating the specificity of circUHRF1 silencing (Additional file 3**: Supplementary Fig. 1b**). Our results also showed that exosomal circUHRF1 expression was significantly decreased in circUHRF1 knockdown cells (Fig. 3h). Interestingly, the reduction in exosomal circUHRF1 levels effectively attenuated HCC-derived exosome-induced impairment of IFN-γ and TNF-α secretion in NK cells (Fig. 3i and j). These results collectively suggest that HCC-derived exosomal circUHRF1 impaired the functions of NK cells.

### CircUHRF1 and miR-449c-5p degrade each other in NK cells

**Fig. 4:**
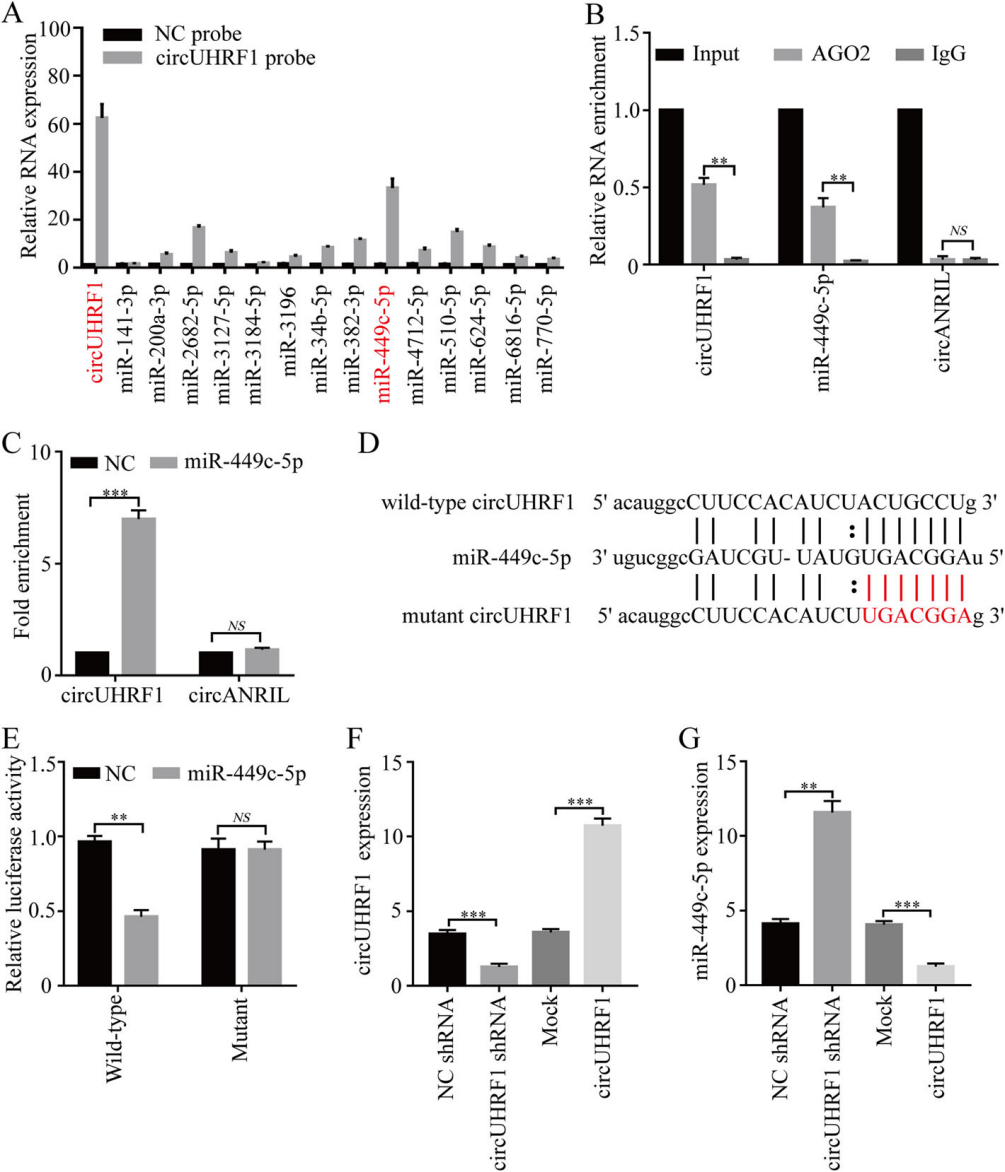
CircUHRF1 inhibits the biological function of miR-449c-5p in NK cells. **a** RIP was performed for circRNA in NK-92 cells using a circUHRF1 probe and a negative control (NC) probe. **b** RIP experiments were carried out using an anti-AGO2 antibody with NK-92 cell extracts. **c** The level of circUHRF1 in the streptavidin-captured fractions of NK-92 cell lysates after transfection with biotinylated miR-449c-5p or the negative control (NC). CircANRIL was used as a negative control. **d** Putative binding sites of miR-449c-5p on circUHRF1 were predicted by StarBase v3.0. **e** The luciferase activity of pGL3-circUHRF1 in NK-92 cells after cotransfection with miR-449c-5p. **f** CircUHRF1 expression in NK-92 cells was modified by lentivirus-mediated overexpression and knockdown. **g** The relative level of miR-449c-5p was measured by qRT-PCR in NK-92 cells transfected with circUHRF1, shcircUHRF1, mock, or the negative control. The data are presented as the mean ± SD; *n* = 3, **P < 0.01, ***P < 0.001, NS: not significant

Previous studies have reported that circRNAs mainly function as miRNA sponges to bind miRNAs and then promote miRNA-targeted gene expression. We next investigated whether circUHRF1 could bind to miRNAs in human NK-92 cells. Through StarBase v3.0 analysis, 14 miRNAs were predicted as possible targets of circUHRF1. To verify the critical miRNAs that may interact with circUHRF1 in NK-92 cells, we used a circUHRF1-specific probe to carry out circRNA in vivo precipitation (circRIP) and detected the 14 predicted miRNAs in complex with circUHRF1 using qRT-PCR. Our results showed a significant enrichment of circUHRF1 and miR-449c-5p compared with the negative controls, while the other miRNAs had no enrichment or only slight enrichment (Fig. 4a), indicating that miR-449c-5p is a circUHRF1-interacting miRNA in NK-92 cells. Given that circRNAs act as miRNA sponges [18, 28, 29], we wondered whether circUHRF1 can interact with certain miRNAs in NK cells. Next, we conducted RIP with an anti-AGO2 antibody in NK-92 cells. The results showed that circUHRF1 and miR-449c-5p, but not circANRIL (a circular RNA reported to not bind to AGO2 [30]), were significantly enriched by the anti-AGO2 antibody (Fig. 4b). This result indicated that circUHRF1 may act as a binding platform for miR-449c-5p and AGO2. To further verify this result, a biotinylated miR-449c-5p pulldown assay was performed, and the results showed significant enrichment of circUHRF1 compared with the negative control in NK-92 cells (Fig. 4c). Next, we carried out a luciferase assay using miR-449c-5p mimics cotransfected with a pGL3 luciferase plasmid expressing the wild-type circUHRF1 sequence or an miR-449c-5p binding site-mutant circUHRF1 sequence into NK-92 cells and normal NK cells (derived from a healthy donor) (Fig. 4d). Compared with the negative control, miR-449c-5p mimics significantly reduced the luciferase reporter activity of the wild-type but not the miR-449c-5p-binding site-mutant pGL3 luciferase plasmid (Fig. 4e; Additional file 3**: Supplementary Fig. 2**). Moreover, the level of circUHRF1 was significantly decreased after overexpression of miR-449c-5p, and the level of miR-449c-5p was significantly decreased after overexpression of circUHRF1 in NK-92 cells (Additional file 3**: Supplementary Fig. 3a and b**; Fig. 3f and g). These findings suggest that circUHRF1 and miR-449c-5p may target each other. All of the above results confirmed that circUHRF1 and miR-449c-5p affected each other in NK cells.

Therefore, we speculated that circUHRF1 may inhibit miR-449c-5p activity to promote functional impairment of NK cells. To test this hypothesis, we ectopically overexpressed circUHRF1 together with miR-449c-5p in NK-92 cells to evaluate the regulation of miR-449c-5p by circUHRF1 (Additional file 3**: Supplementary Fig. 4a**). Although forced miR-449c-5p expression alone showed significant tumor-suppressive activity (promoting IFN-γ and TNF-α secretion), increased circUHRF1 expression dramatically inhibited the miR-449c-5p tumor-suppressive function (Additional file 3**: Supplementary Fig. 4b**), highlighting the critical ability of HCC-derived exosomal circUHRF1 to inhibit the function of NK cells via the miR-449c-5p-related pathway.

### CircUHRF1 upregulates TIM-3 expression via suppression of miR-449c-5p activity

**Fig. 5:**
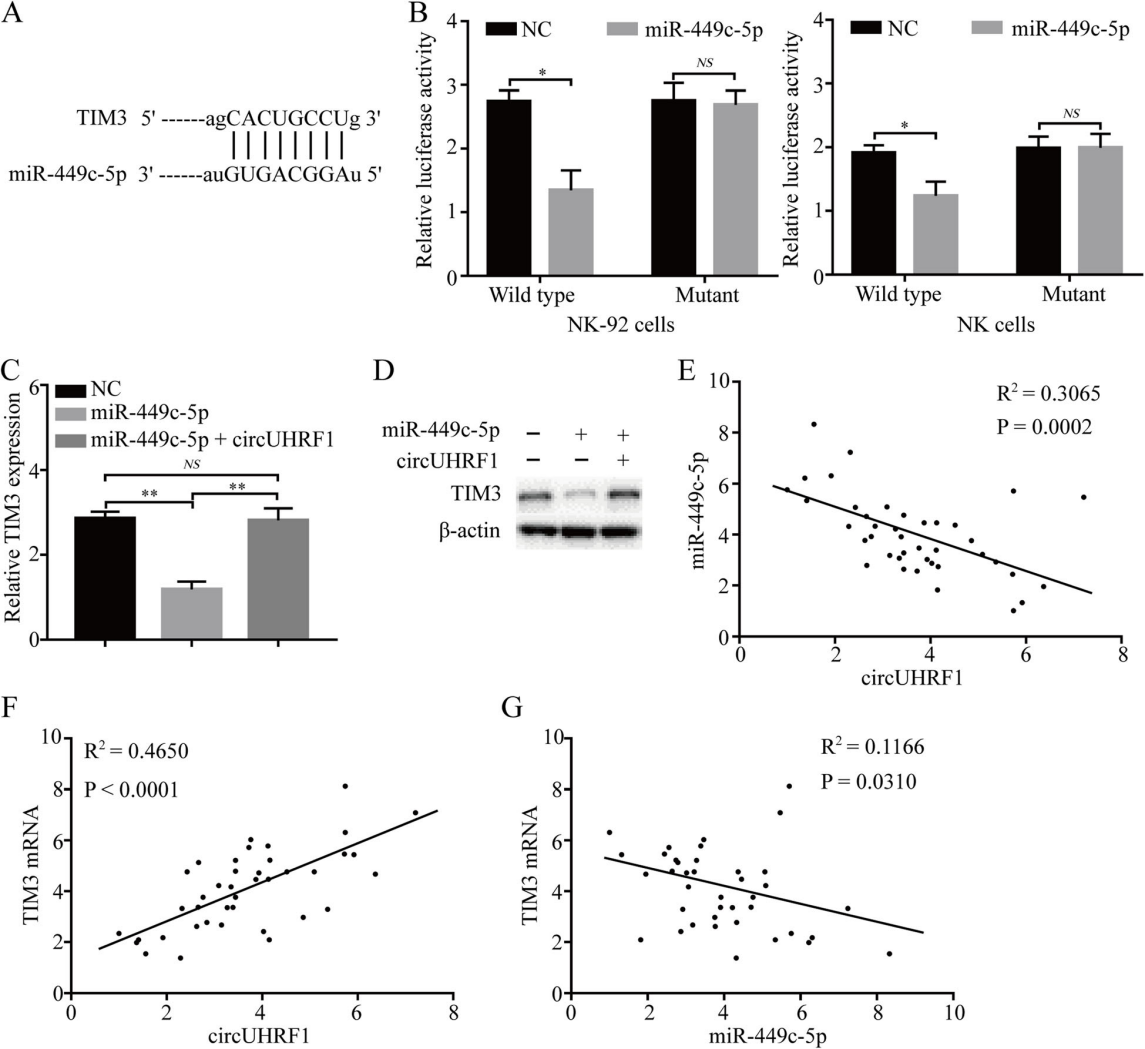
CircUHRF1 regulates the miR-449c-5p/ TIM-3 pathway in NK cells. **a** The putative binding site of miR-449c-5p on TIM-3 was predicted by StarBase v3.0. **b** Luciferase activity of pGL3-TIM-3 in NK-92 cells cotransfected with miR-449c-5p. **c and d** The levels of TIM-3 mRNA and protein were measured by qRT-PCR and western blotting, respectively, in NK-92 cells transfected with miR-449c-5p or miR-449c-5p combined with circUHRF1. **e** A negative correlation between circUHRF1 and miR-449c-5p expression was observed in NK cells from HCC patients (*R*^2^ = 0.3065; *P* = 0.0002). **f** A positive correlation between circUHRF1 and TIM-3 mRNA expression was observed in NK cells from HCC patients (*R*^2^ = 0.4650; *P* < 0.0001). **g** A negative correlation between miR-449c-5p and TIM-3 mRNA expression was observed in NK cells from HCC patients (*R*^2^ = 0.1166; *P* = 0.0310). The data are presented as the mean ± SD; *n* = 3, *P < 0.05, **P < 0.01, NS: not significant

As our data showed that circUHRF1 effectively inhibited the function of miR-449c-5p to suppress NK cell secretion of IFN-γ and TNF-α, we speculated that circUHRF1 may be responsible for upregulating the expression levels of miR-449c-5p targets by acting as a miR-449c-5p sponge and enhancing immune evasion in HCC patients. To verify this hypothesis, we first analyzed a panel of predicted miR-449c-5p target genes in NK cells, which included TIM-3 (Fig. 5a). Next, we carried out a luciferase reporter assay. The pGL3 luciferase plasmid was created based on firefly luciferase expressing the wild-type 3′ UTR of TIM-3 or 3′ UTR TIM-3 sequence with a mutated miR-449c-5p-binding site. NK-92 cells and healthy donor NK cells were cotransfected with the pGL3 luciferase plasmid along with negative control or miR-449c-5p mimics and assayed for luciferase activity. Compared with the negative control, miR-449c-5p mimics significantly reduced the luciferase reporter activity of wild-type TIM-3 (Fig. 5b). Interestingly, we verified that TIM-3 expression was significantly decreased after forced expression of miR-449c-5p (Fig. 5c and d).

In light of the critical role of TIM-3-related pathways in immune evasion, we hypothesized that circUHRF1 may be an important contributor to HCC immune evasion via its competitive circRNA activity. Our results showed that TIM-3 mRNA is a target of miR-449c-5p in NK cells. Therefore, continuous upregulation of exosomal circUHRF1 sequesters miR-449c-5p in NK cells and thereby leads to immune evasion in HCC. To further validate our hypothesis, we detected TIM-3 expression in NK-92 cells transfected with miR-449c-5p mimic alone or together with circUHRF1. The results showed that forced miR-449c-5p expression led to decreased mRNA and protein expression levels of TIM-3, while circUHRF1 reversed this inhibitory effect of miR-449c-5p on TIM-3 expression (Fig. 5b and c).

To further confirm our in vitro results that circUHRF1 impeded NK cell function through regulation of the miR-449c-5p/TIM-3 axis, we investigated the correlation between circUHRF1 and miR-449c-5p/TIM-3 expression in another 40 HCC patient peripheral NK cells. We found that circUHRF1 expression was negatively correlated with miR-449c-5p expression in peripheral NK cells (*R*^2^ = 0.3065; *P* = 0.0002; Fig. 5e). As expected, we also found that circUHRF1 expression positively correlated with TIM-3 mRNA expression in peripheral NK cells (*R*^2^ = 0.4650; *P* < 0.0001; Fig. 5f). Notably, we observed that miR-449c-5p expression was negatively associated with the expression of TIM-3 mRNA in peripheral NK cells (*R*^2^ = 0.1166; *P* = 0.0310; Fig. 5g), indicating that the upregulation of exosomal circUHRF1 abrogates the tumor-suppressive effect of miR-449c-5p on TIM-3 in NK cells.

Previous studies have reported that both NK cells and CD8^+^ T cells infiltrating tumors have increased TIM-3 expression [31–33]. Therefore, we performed experiments to explore whether HCC-derived exosomal circUHRF1 inhibits the function of CD8^+^ T cells. Regrettably, compared with CD8^+^ T cells cultured alone, CD8^+^ T cells cocultured with SMMC-7721 and HCCLM3 cells demonstrated no significant impairment of IFN-γ and TNF-α secretion when subsequently cocultured with K562 cells (**Supplementary Fig.****5****a and b**). In addition, CD8^+^ T cells cocultured with SMMC-7721 and HCCLM3 cells demonstrated significantly increased circUHRF1 expression (**Supplementary Fig.****5****c**). By determining miR-449c-5p expression in CD8^+^ T and NK cells, we found that the difference in the abundance of miR-449c-5p was obvious, and was higher in NK cells than in CD8^+^ T cells (**Supplementary Fig.****5****d**). These results might be indicate that the circUHRF1/miR-449c-5p/TIM-3 axis is specific to NK cells and does not function in CD8^+^ T cells.

### CircUHRF1 induces HCC progression in an NK cell-dependent manner

**Fig. 6:**
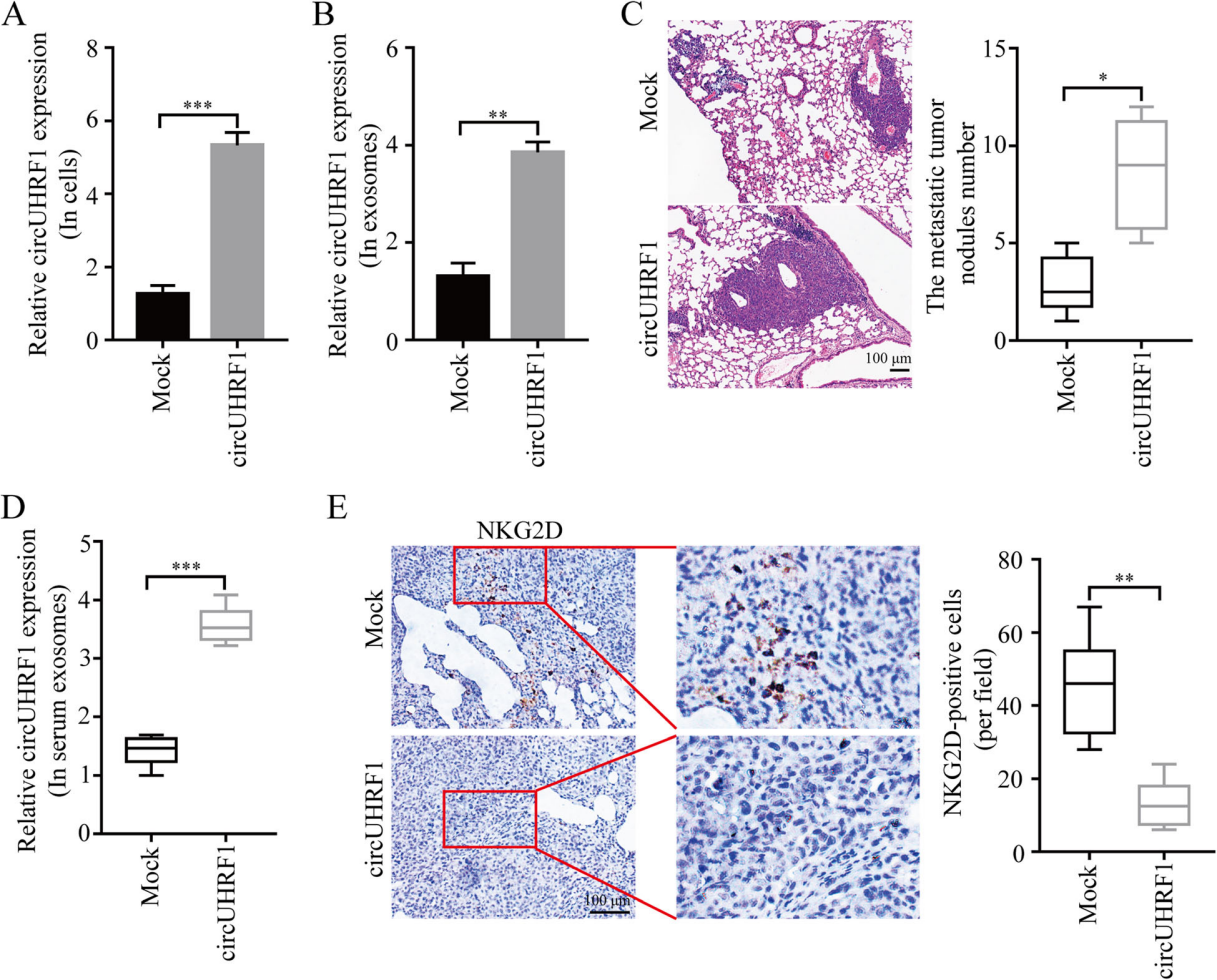
CircUHRF1 induces immune evasion in an NK cell-dependent manner. **a** CircUHRF1 expression in PLC/PRF/5 cells was modified by lentiviral vector transfection. **b** CircUHRF1 expression in HCC cell-derived exosomes was increased in the PLC/PRF/5-circUHRF1 group. **c** The effects of circUHRF1 overexpression on lung metastasis were investigated in vivo (*n* = 6 mice for each group). **d** CircUHRF1 expression in mouse serum-derived exosomes was increased in PLC/PRF/5-circUHRF1 cells. **e** NKG2D-positive cells in the PLC/PRF/5-circUHRF1 or PLC/PRF/5-mock cell-derived tissues were analyzed by IHC. The data are represented as the mean ± SD; *n* = 3, *P < 0.05, **P < 0.01, ***P < 0.001

To further explore the function of circUHRF1 in HCC, we used the pLO5-ciR-circUHRF1 lentivirus vector to upregulate the expression of circUHRF1 in PLC/PRF/5 cells. qRT-PCR results showed that the circUHRF1 levels in PLC/PRF/5 cells transfected with pLO5-ciR-circUHRF1 were significantly higher than those in the cells transfected with pLO5-ciR-Mock (Fig. 6a). Importantly, exosomes derived from HCC cells transfected with pLO5-ciR-circUHRF1 had higher levels of circUHRF1 than those derived from pLO5-ciR-Mock-transfected HCC cells (Fig. 6b). Next, we generated a NOD/SCID mouse model (injected with NK cells derived from healthy donors) of tumor lung metastasis using PLC/PRF/5 cells. The results showed that the number of metastatic tumor nodules induced by PLC/PRF/5 cells transfected with pLO5-ciR-circUHRF1 was significantly increased compared with that induced by HCC cells transfected with pLO5-ciR-Mock (Fig. 6c). Importantly, the mice with tumor lung metastases derived from PLC/PRF/5 cells with forced circUHRF1 expression showed an increased circUHRF1 level in serum exosomes (Fig. 6d). Additionally, the number of NKG2D-positive cells in metastatic tumor nodules induced by HCC cells transfected with pLO5-ciR-circUHRF1 was significantly decreased compared with that in tumors induced by HCC cells transfected with pLO5-ciR-Mock (Fig. 6e). These results indicate that circUHRF1 promotes HCC progression in an exosome- and NK cell-dependent manner.

### CircUHRF1 enhances HCC resistance to anti-PD1 therapy

**Fig. 7:**
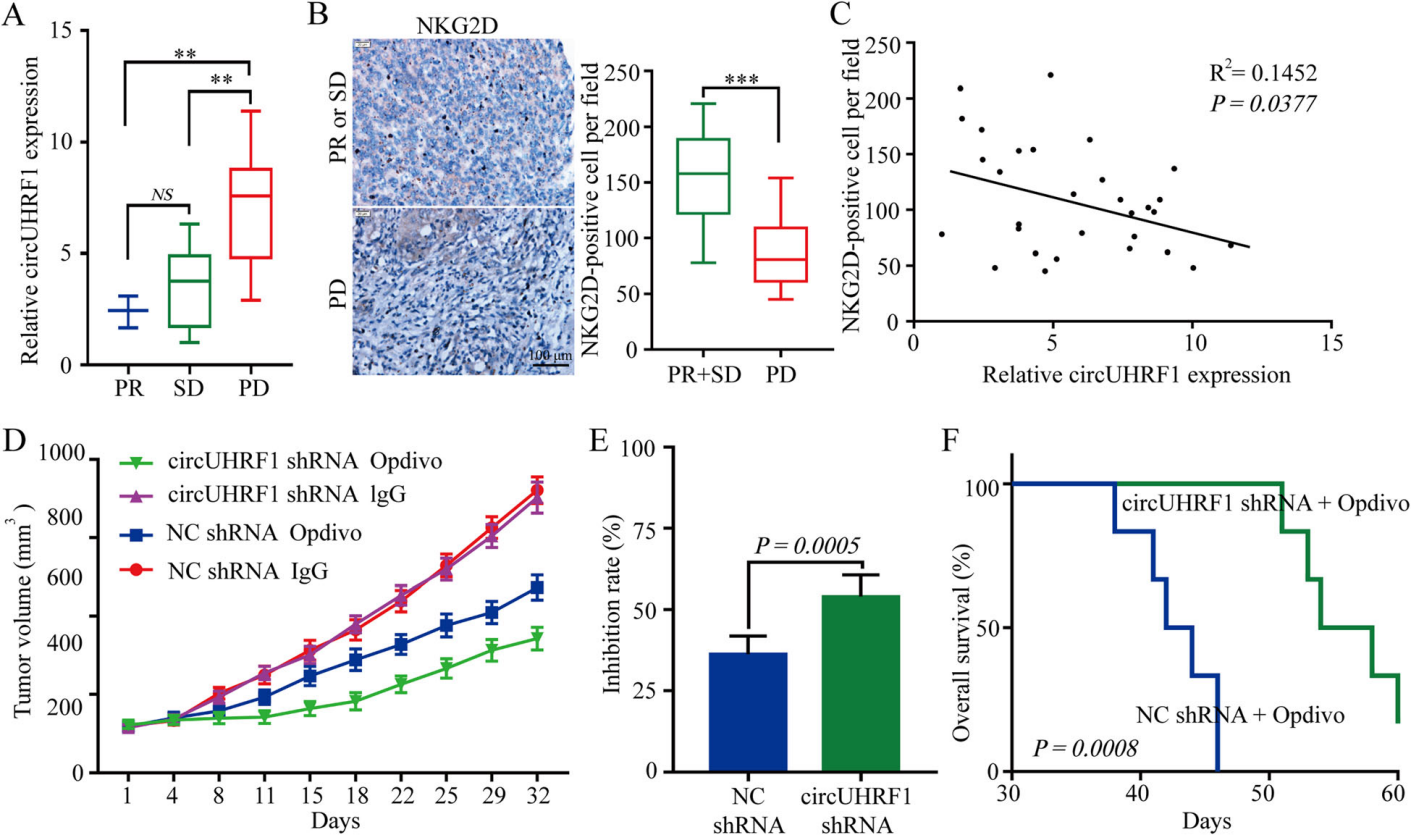
Higher levels of circUHRF1 correlate with resistance to anti-PD1 therapy in HCC patients. **a** Thirty patients were divided into PR, SD, and PD groups. The diagram shows circUHRF1 expression in each group. **b** Representative HCC cases were analyzed by IHC staining for NKG2D. **c** A negative correlation between miR-449c-5p and the number of NKG2D-positive cells was observed in the HCC tissues (*R*^2^ = 0.1452; *P* = 0.0377). **d** HCCLM3-NC shRNA or HCCLM3-circUHRF1 shRNA cells were subcutaneously injected into 4- to 6-week-old NOD/SCID mice, and when tumors reached a mean tumor volume of 100 mm^3^, NK (10^6^) cells were injected intravenously through the tail vein, and mice were treated with IgG or Opdivo. The data are expressed as the mean tumor volume (the data are presented as the mean ± SD; *n* = 6). **e** The data are expressed as the percent of tumors with inhibited growth (the data are presented as the mean ± SD; *n* = 6). **f** Comparison of the overall survival curves for mice with high and low circUHRF1 expression in xenograft HCC tumors that were treated with Opdivo. **P < 0.01, ***P < 0.001. NS: not significant

Because forced circUHRF1 expression suppresses NK cell function in HCC, we next evaluated whether circUHRF1 overexpression can further impede the antitumor effect of anti-PD1 therapy (Opdivo). Therefore, we analyzed retrospective data from 30 HCC patients with recurrent distant metastasis (including lung metastasis) receiving anti-PD1 immunotherapy who had undergone liver resection 2–36 months before immunotherapy. After six therapy cycles, the efficacy was assessed using CT. Based on RECIST1.1 analysis, the results showed that there were 3 patients with partial response (PR), 7 patients with stable disease (SD), and 20 patients with progressive disease (PD) (Additional file 3**: Supplementary Fig. 6**). Then, circUHRF1 expression levels were measured, and the results indicated that the circUHRF1 expression levels in the PD group were much higher than those in the SD and PR groups (Fig. 7a). To further explore the relationship between circUHRF1 and immune evasion, we examined the expression of NKG2D in tissues from 30 HCC patients. The number of NKG2D-positive cells in HCC patients resistant to anti-PD1 therapy was significantly reduced compared with that in HCC patients susceptible to anti-PD1 therapy (Fig. 7b). A scatter plot analysis revealed a negative correlation between circUHRF1 expression and NKG2D-positive cell number in HCC tissues (*R*^2^ = 0.1452; *P* = 0.0377) (Fig. 7c). To further assess the effects of circUHRF1 on resistance to anti-PD1 therapy, we established a xenograft model by subcutaneous implantation of circUHRF1-knockdown HCCLM3 cells or the respective negative control cells. The results indicated that the implantation of circUHRF1 knockdown cells resulted in sensitivity to anti-PD1 treatment and an increase in the overall survival rate (Fig. 7d-f). Taken together, these findings indicate that forced circUHRF1 expression might impede the response of HCC to anti-PD1 treatment and that targeting circUHRF1 might be a promising and effective method to recover the sensitivity of HCC to anti-PD1 therapy.

## Discussion

In recent years, numerous studies have verified the abnormal expression of circRNAs in a series of cancers and have revealed that many circRNAs play vital roles in modulating tumor immunosuppression, proliferation, migration, invasion, metastasis, and chemotherapy resistance [24, 34, 35]. However, the biological molecular mechanisms by which circRNAs participate in cancer remain unclear [6, 36]. Here, we report a UHRF1-derived circular RNA and evaluated its biological functions in promoting HCC immunosuppression. We found that circUHRF1 was highly expressed in HCC tissues and HCC-derived exosomes. Furthermore, we demonstrated that HCC-secreted exosomal circUHRF1 was delivered into NK cells, upregulated TIM-3 expression by sponging miR-449c-5p and in turn induced NK cell exhaustion. More importantly, we found that circUHRF1-overexpressing HCC cells were characterized by resistance to anti-PD1 treatment in nude mice adoptively transferred with NK cells. This result was also proven by a retrospective study that analyzed a cohort of 30 HCC patients treated with anti-PD1 agents. All of the above results demonstrated that exosomal circUHRF1 secreted by HCC cells mediates resistance to anti-PD1 therapy by inducing NK cell exhaustion, which may provide a potential therapeutic strategy for patients with HCC.

Increasingly, studies have reported that intercellular communication plays a critical role in promoting the immunosuppression, proliferation, migration, and invasion of cancers [37–40]. Direct interactions, secreted biologically active molecules and exosomes are the major mechanisms for cell signal exchange [39, 40]. Usually, exosomes reflect the malignant features of donor cells and transport oncogenic signals to recipient cells that can promote cancer progression. Here, our results highlight a critical role for exosomal circUHRF1 in inducing immune evasion and resistance to anti-PD1 therapy by inducing NK cell dysfunction in HCC.

Recently, an increasing number of studies have verified exosomal circRNAs in the peripheral blood of patients with a variety of cancers, including HCC [39, 41, 42]. Importantly, circRNAs have been confirmed to participate in the regulation of various immune responses, including cancer immune evasion [34]. Our results on immune evasion and the tumor progression-promoting effects of exosomal circUHRF1 are likely to be relevant in HCC. Identifying patients with higher levels of exosomal circUHRF1 will be critical in predicting those who are more likely to be resistant to anti-PD1 immunotherapy.

NK cells have been verified to have cytotoxic effects against tumor cells in several cancers, including HCC [43–45]. PD1 is an inhibitory receptor expressed on activated lymphocytes, including T cells, NK cells, and B cells [46]. PD1 can be overexpressed on NK cells from HCC patients and promotes functional dysregulation of activated NK cells [47, 48]. Current research has reported that decreased NK cell infiltration in tumor tissue predicts resistance to anti-PD1 immunotherapy [49, 50]. TIM-3 is an important coinhibitory molecule expressed on T cells and NK cells. Compared with NK cells from healthy donors, peripheral NK cells from cancer patients express significantly higher levels of surface TIM-3 [31]. Importantly, increased TIM-3 expression is associated with functional impairment and death of NK cells [31]. Previous studies have also reported that TIM-3 expression on NK cells from cancer patients transmits negative signals of NK cell cytotoxicity [32, 51]. In this study, our results showed that the expression of circUHRF1 was significantly increased not only in HCC tissues but also in HCC-derived exosomes. Furthermore, we found that higher expression of circUHRF1 was dramatically associated with poor prognosis and pathological characteristics, suggesting its tumor-promoting effect on HCC. Functionally, we demonstrated that HCC-derived exosomes suppress the ability of NK cells to produce IFN-γ and TNF-α via upregulation of TIM-3 expression. In circUHRF1-overexpressing HCC cells, increased intercellular communication between NK cells and HCC cells is likely to bypass the upregulation of TIM-3, resulting in the impaired function and an exhausted phenotype of NK cells.

The unique ability of NK cells to target cancer cells without antigen specificity makes them an ideal candidate for use against those tumors [52]. Currently, the TIM-3/Gal-9 pathway has been well demonstrated in NK cells, and several studies have detected PD1 expression on NK cells in various clinical settings; thus, PD1/PD-L1 and TIM-3/Gal-9 blockade might favor NK cell activity in antitumor immunity [53]. In this study, we showed that HCC-derived exosomal circUHRF1 can upregulate TIM-3 expression, which further induces the NK cell exhaustion. Thus we revealed that circUHRF1 levels are an important factor influencing resistance to anti-PD1 therapy in a subgroup of HCC patients.

Both NK cells and CD8^+^ T cells infiltrating tumors had increased TIM-3 expression, which indicated that anti-TIM-3 agents, as a monotherapy, might be a promising method for reversing the exhaustion of both NK cells and CD8^+^ T cells [31–33]. Here, we found that reduced circUHRF1 expression increased the therapeutic efficacy of anti-PD1 treatment via the exosomal circUHRF1/miR-449c-5p/TIM-3 pathway. TIM-3 expression on tumor-infiltrating NK Cells and CD8^+^ T cells exerts immunosuppressive effects. Anti-TIM-3 agents, as a monotherapy, might lead to benefits to patients outcomes by reversing the exhaustion of both NK cells and CD8^+^ T cells.

## Conclusions

Overall, we have demonstrated in this study that HCC-derived exosomes suppress the production of IFN-γ and TNF-α via the induction of TIM-3 expression on NK cells. We also report a new mechanism by which tumor exosomes inhibit NK cell function via exosomal circUHRF1, contributing new evidence for the crosstalk between HCC cells and NK cells.

## Supplementary information

**Additional file 1.**

**Additional file 2.**

**Additional file 3.**

## Data Availability

All data generated or analyzed during this study are included either in this article or in the supplementary information files.

## Abbreviations

HCC: Hepatocellular carcinoma
OS: Overall survival
RFS: Recurrence-free survival
NKG2D: Natural-killer group 2 member D
TIM-3: T cell immunoglobulin and mucin domain 3
NK cell: Natural killer cell
qRT-PCR: Quantitative real-time polymerase chain
RIP: RNA immunoprecipitation
AGO2: Argonaute 2
UHRF1: Ubiquitin like with PHD and ring finger domains 1
PD1: Programmed cell death 1
CircRNA: Circular RNA
CCK-8: Cell Counting Kit-8
IHC: Immunohistochemistry
TMA: Tissue microarray
