# Supplementary material for: Cancer cell-derived exosomal circUHRF1 induces natural killer cell exhaustion and may cause resistance to anti-PD1 therapy in hepatocellular carcinoma
Source: Mol Cancer. 2020 Jun 27;19:110. doi: 10.1186/s12943-020-01222-5 (PMC7320583; doi:10.1186/s12943-020-01222-5)
Supplement: Supplementary file 1 — Additional file 1. [file 12943_2020_1222_MOESM1_ESM.docx]

**Supplementary Methods**

***Clinical tissues***

Clinical tissues, blood, tumors, and areas adjacent to the margin of tumors, were collected from patients with HCC who underwent curative resection between 2010 and 2013 at the Liver Cancer Institute of Fudan University (Shanghai, China). Parafﬁn blocks were selected only on the basis of the availability of suitable formalin-ﬁxed, parafﬁn-embedded tissue and complete clinicopathologic and follow-up data for the patients. The histopathological diagnosis was based on the World Health Organization (WHO) criteria. Ethical approval was obtained from the Zhongshan Hospital Research Ethics Committee, and written informed consent was obtained from all of the patients (permit ID number: Y2016-025). The patients with disease relapse were confirmed via enhanced CT or enhanced MRI, and the serum exosomes were collected in early relapse stage.

***qRT-PCR and western blotting analysis***

Total RNA was extracted using TRIzol (Invitrogen, USA, Cat: 15596026) and reverse-transcribed to cDNA using a PrimeScript RT Reagent Kit (Takara, Japan, Cat: RR037Q) according to the manufacturer’s instructions. Gene amplification and detection were performed using the ABI PRISM 7900 Sequence Detection System (Applied Biosystems, Foster City, CA, USA, Cat: 4317596) and SYBR Green Real-time PCR Master Mix Kit (Takara, Japan, Cat: DRR041A). All transcript levels were normalized to glyceraldehyde 3-phosphate dehydrogenase (GAPDH) expression. The relative expression was analyzed by the comparative cycle threshold (Ct) method, according to the equation 2^-ΔCt^ [ΔCt = Ct-Ct (GAPDH)]. GAPDH also as an internal reference for exosomal circUHRF1. All experiments were performed in triplicate.

For western blotting, the total protein extracts from cells were separated by sodium dodecyl sulfate-polyacrylamide gel electrophoresis (SDS-PAGE), transferred onto polyvinylidene difluoride membranes, and incubated with the corresponding antibodies. The membranes were developed using the enhanced chemiluminescence method (Pierce, Rockford, IL, USA, Cat: 32109).

***In vivo tumor growth and metastasis assays***

Xenograft experiments in NOD/SCID mice were approved by the Animal Experimentation Ethics Committee of Zhongshan Hospital, Fudan University (permit ID number: Y2016-025). Male NOD/SCID mice aged 4-6 weeks were maintained according to the stated guidelines of the 3 Rs (replacement, reduction, and refinement). We resuspended 10^5^ cells (per mouse) in 100 μl of PBS and injected them into the lateral tail vein. After 30 days, we resuspended 10^6^ NK-92 cells (per mouse) in 100 μl of PBS and injected them into the lateral tail vein. The mice were sacrificed after 35 days; the lungs were resected, embedded in paraffin, and stained with hematoxylin and eosin (H&E); and lung metastases and NK cell infiltration were evaluated. Infiltrating NK cells positively stained for NKG2D in each TMA core were counted manually by two independent pathologists under high-power magnification (200×).

***Statistical analysis***

In brief, Student’s t test or Tukey’s multiple comparisons test was used for comparisons between two groups, and one-way ANOVA was used for multiple group comparisons. The cumulative recurrence and survival rates were analyzed using Kaplan-Meier’s method and the log-rank test. Cox’s proportional hazard regression model was used to analyze independent prognostic factors. The categorical data were analyzed by chi-square or Fisher’s exact tests. Correlation analysis was performed for NK cell proportion in blood, circUHRF1, miR-449c-5p, TIM-3, and NKG2D.
