# Supplementary material for: Cancer cell-derived exosomal circUHRF1 induces natural killer cell exhaustion and may cause resistance to anti-PD1 therapy in hepatocellular carcinoma
Source: Mol Cancer. 2020 Jun 27;19:110. doi: 10.1186/s12943-020-01222-5 (PMC7320583; doi:10.1186/s12943-020-01222-5)
Supplement: Supplementary file 2 — Additional file 2. [file 12943_2020_1222_MOESM2_ESM.docx]

Supplementary Table 1. The qRT-PCR primers used in this study.

| **Gene** | **Forward primer (5’-3’)** | **Reverse primer(5’-3’)** |
| --- | --- | --- |
| hsa_circ_0002185 | TGGGGCTGACCATGCAGGGCG | ACCTTGTAGATGCCATCGTAG |
| hsa_circ_0002694 | GCCAACGTGGTGCTGGGCAGC | CGTGGAGCTGCAGGGCTCGTC |
| hsa_circ_0002838 | GGCGGTTCCGAGTCCAGGTAC | ATGGGTCCGTAGTGGTTGGAC |
| hsa_circ_0005083 | CTCACCAACACCAACAGGGAT | ACGTCGTCCTTGCAGTGCTTG |
| hsa_circ_0005837 | CCCAGCGAGGACGAGTGGGAT | CATGGCCTTCCACATCTACTG |
| hsa_circ_0009107 | CTCACCAACACCAACAGGTAC | ACGATGGTACATTCCTTGGTG |
| hsa_circ_0048672 | TGGGGCTGACCATGCAGGGCC | AGGCATTAACGACTAGCCAAG |
| hsa_circ_0048673 | AGCACAACGTGTGCAAGCGCC | GGTATGGCCGTCCTCCATCTG |
| hsa_circ_0048674 | AATTGGGGCTGTACAAGATGG | GGACTTGTCTGACTCACTCTG |
| hsa_circ_0048675 | CACGTGAAATACGACGAATGG | GGACTTGTCTGACTCACTCTG |
| hsa_circ_0048676 | TGGGGCTGACCATGCAGGTCA | GTGGTAAATGACGTCCTCCTC |
| hsa_circ_0048677 | GCTATGAGGATGATGTGGGAT | CAGAGTCTGTTCACGTCGTCC |
| hsa_circ_0048678 | GGCGGTTCCGAGTCCAGGGCA | ACATGGTGCCCACGGGGATCC |
| hsa_circ_0048679 | CGGCGAGCGGCAGCCCGTATC | TGCTGGGCCGTGAGACTGTAG |
| UHRF1 | ATGACACCATCCAGCTCCTGG | CTCATCCCACATGTCCTCATC |
| TIM-3 | CATCAAACCAGCCAAGGTCAC | CTGATGGTTGCTCCAGAGTCC |
| GAPDH | GGGGCTCTCCAGAACATCATCC | ACGCCTGCTTCACCACCTCTT |

Supplementary Table 2. Antibody for western blotting, RIP, and immunohistochemistry.

| **Antibody** | **Company** | **Cat No.** |
| --- | --- | --- |
| NKG2D | Abcam | ab203353 |
| TIM-3 | Abcam | ab185703 |
| β-actin | Abcam | ab8226 |
| CD8 | Abcam | ab4055 |
| PD-L1 | Abcam | ab213480 |
| PD1 | Abcam | ab214421 |
| HRP-labeled Goat Anti-Rabbit IgG(H+L) | Beyotime | A0208 |
| HRP-labeled Goat Anti-mouse IgG(H+L) | Beyotime | A0216 |
| AGO2 | Abcam | ab32381 |
| IgG | Abcam | ab172730 |

Supplementary Table 3. The sequence of circUHRF1 probe.

| Probe | Sequence |
| --- | --- |
| circUHRF1 | AGTCGTTCAGAGAATCATCCCACATCATCCTCATAGCCCCCCGCCAGGACTAGGG |

Supplementary Table 4. Target sequences of circUHRF1 shRNA.

| **shRNA** | **Target sequence** |
| --- | --- |
| shRNA -1 | GAGGATGATGTGGGATGAT |
| shRNA -2 | GATGTGGGATGATTCTCTG |
| shRNA -3 | GTGGGATGATTCTCTGAAC |
