# Supplementary material for: Cancer cell-derived exosomal circUHRF1 induces natural killer cell exhaustion and may cause resistance to anti-PD1 therapy in hepatocellular carcinoma
Source: Mol Cancer. 2020 Jun 27;19:110. doi: 10.1186/s12943-020-01222-5 (PMC7320583; doi:10.1186/s12943-020-01222-5)
Supplement: Supplementary file 3 — Additional file 3. [file 12943_2020_1222_MOESM3_ESM.docx]

**Supplementary Figures and Figure legends**

**Supplementary Figure 1.**


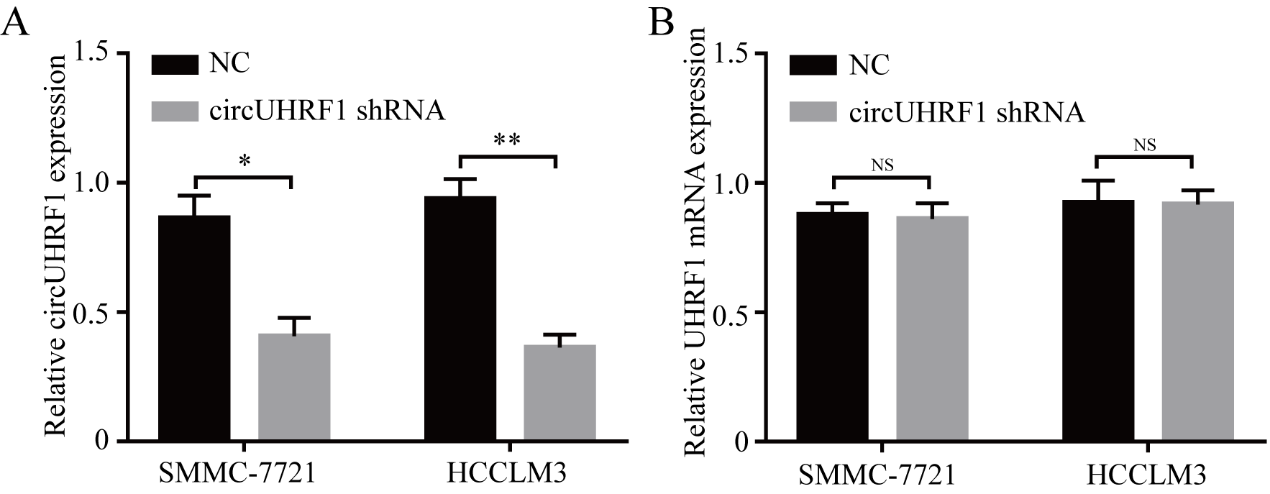


**Supplementary Fig. 1. CircUHRF1 expression in HCC cells was modified by a specific shRNA.** **a** circUHRF1 expression in SMMC-7721 and HCCLM3 cells was knocked down by transfection of shRNA. **b** UHRF1 mRNA expression in circUHRF1-knockdown HCC cells. The data are presented as the mean ± SD. *P < 0.05; **P < 0.01; NS, not significant.

**Supplementary Figure 2.**


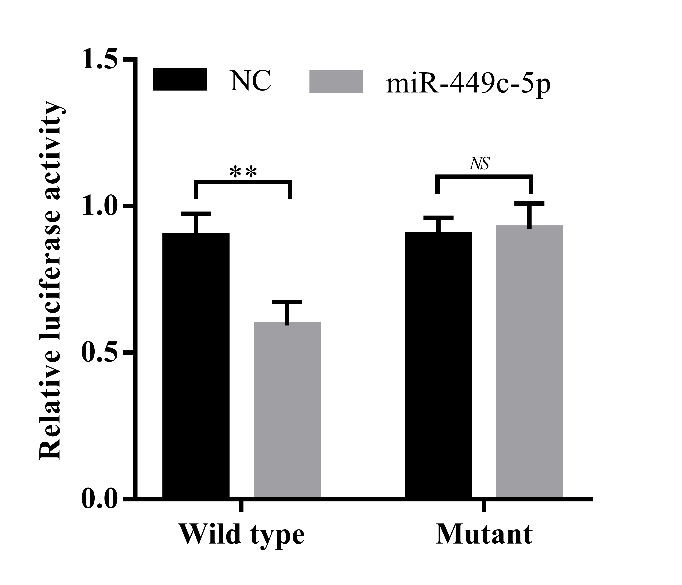


**Supplementary Fig. 2.** The luciferase activity of pGL3-circUHRF1 in NK cells from healthy donor after cotransfection with miR-449c-5p.

**Supplementary Figure 3.**


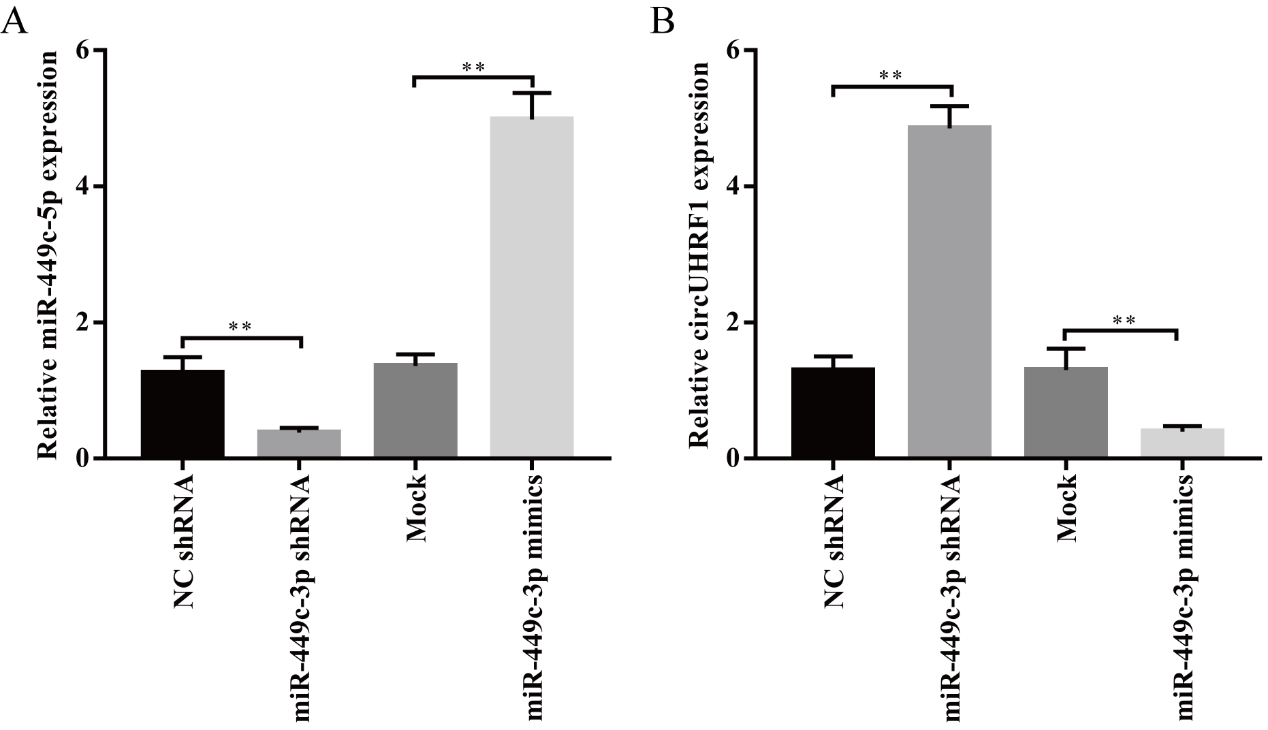


**Supplementary Fig. 3. The relationship between circUHRF1 and miR-449c-5p expression in NK-92 cells**. **a** miR-449c-5p expression in NK-92 cells was modified by miR-449c-5p mimics or shRNA transfection. **b** circUHRF1 expression in miR-381-3p-overexpressing or miR-381-3p-silenced NK-92 cells. The data are presented as the mean ± SD. **P < 0.01.

**Supplementary Figure 4.**


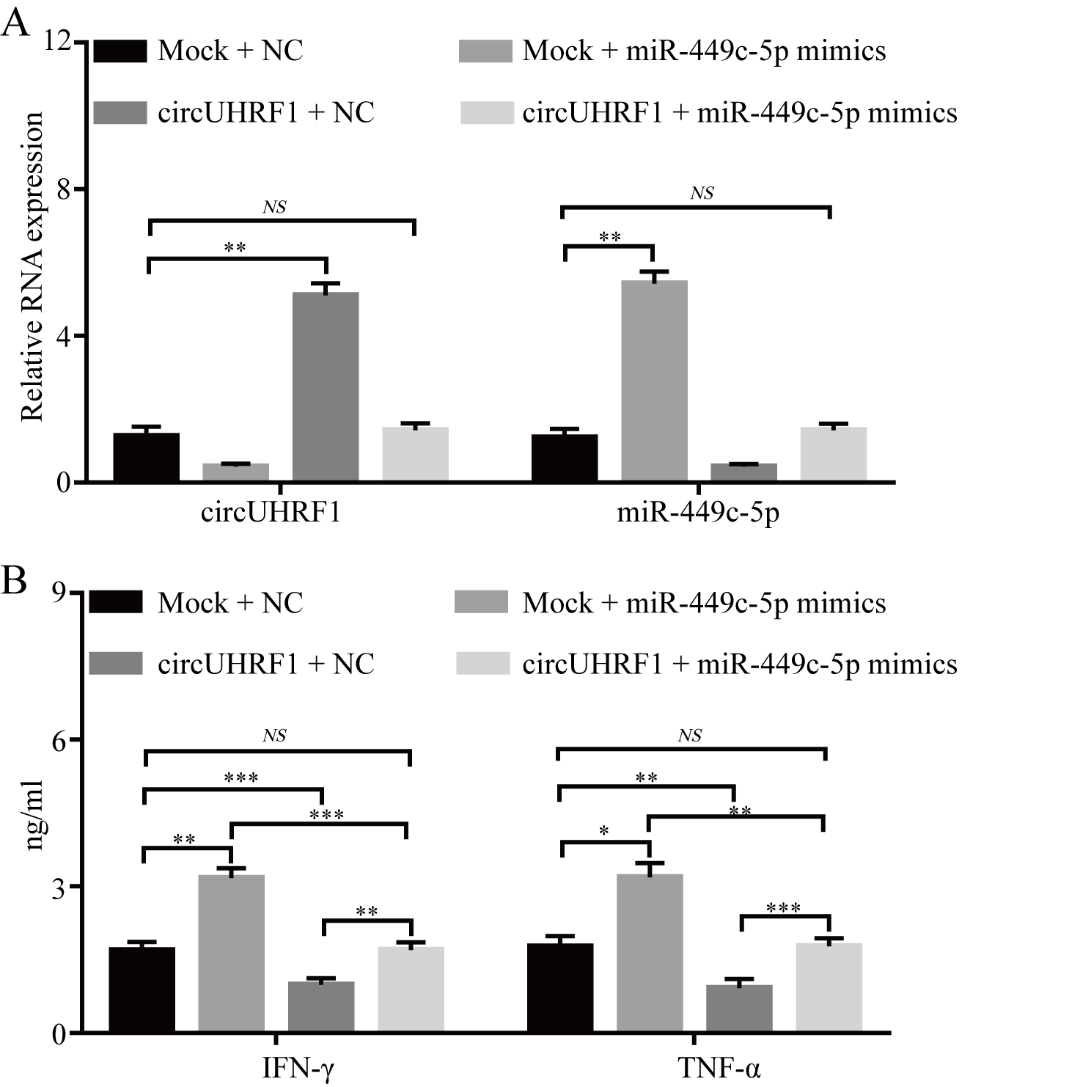


**Supplementary Fig. 4. miR-449c-5p affects the function of circUHRF1 in NK-92 cells. a** The expression of circUHRF1 and miR-449c-5p in NK-92 cells with modified circUHRF1 and miR-449c-5p expression. **b** The secretion of IFN-γ and TNF-α and miR-449c-5p expression in NK-92 cells with modified circUHRF1 and miR-449c-5p expression.

**Supplementary Fig. 5.**


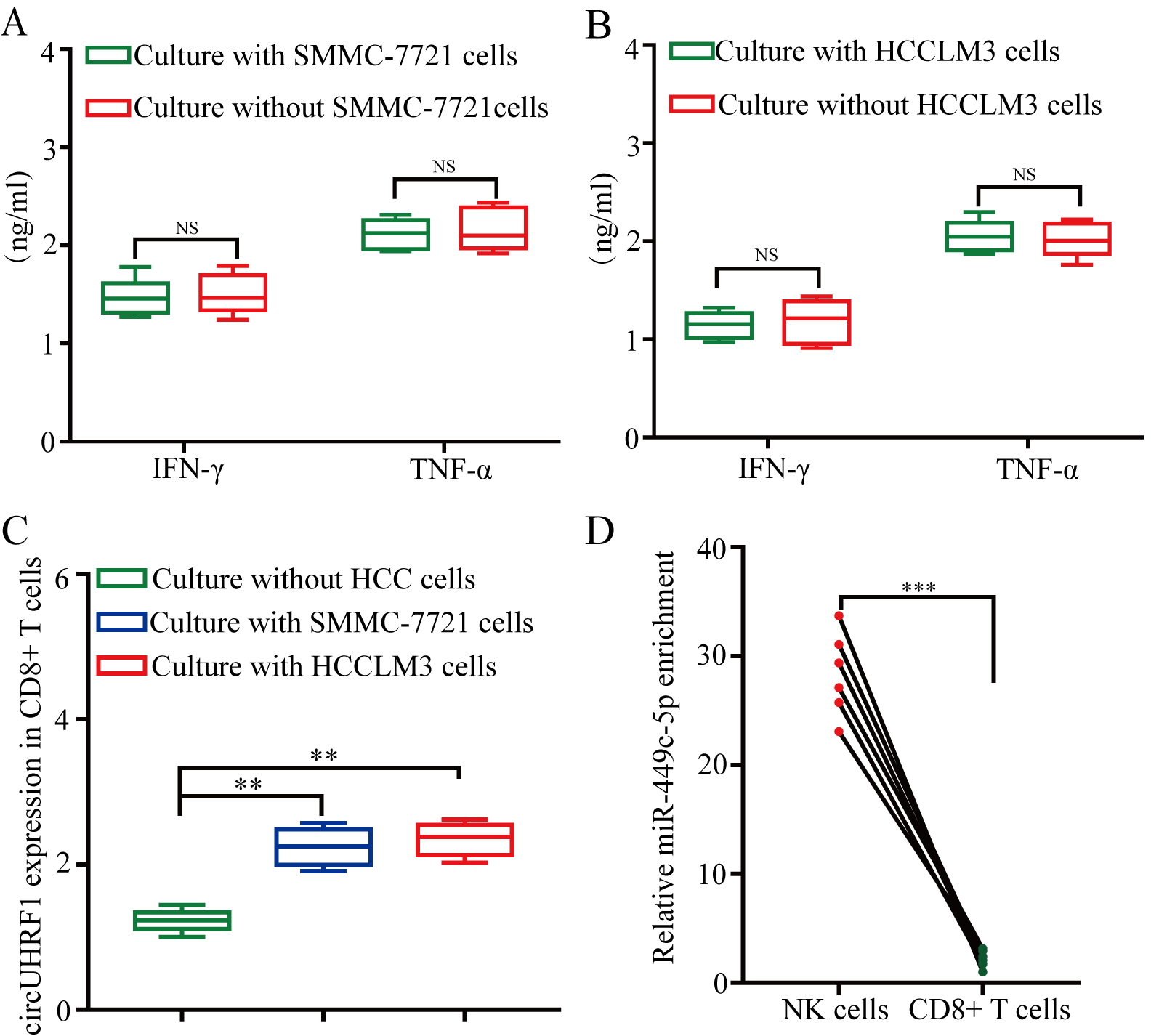


**Supplementary Fig. 5. Exosomal expression of circUHRF1 did not inhibits the function of CD8+ T cells. a and b** HCC cells inhibit CD8+ T cell secretion of IFN-γ and TNF-α. **c** HCC cells upregulate the expression of circUHRF1 in CD8+ T cells. **d**. MiR-449c-5p expression in NK cells and CD8+ T cells was measured by qRT-PCR analysis. The data are presented as the mean ± SD, n = 3, ***P < 0.001.

**Supplementary Fig. 6.**


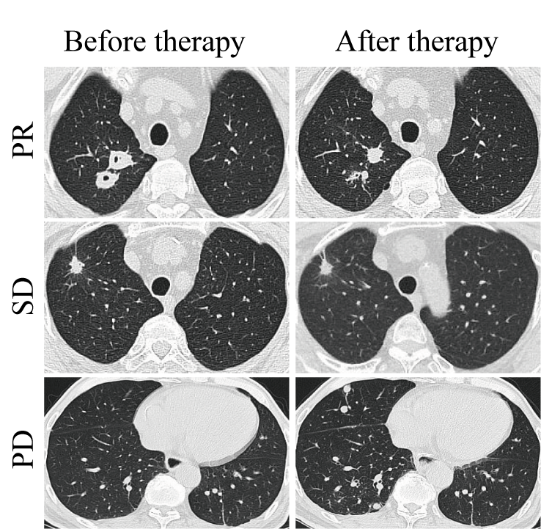


**Supplementary Fig. 6. The PD1 antibody immunotherapy efficacy assessment using CT-based RECIST1.1.**
